# Perception Understanding Action: Adding Understanding to the Perception Action Cycle With Spiking Segmentation

**DOI:** 10.3389/fnbot.2020.568319

**Published:** 2020-10-19

**Authors:** Paul Kirkland, Gaetano Di Caterina, John Soraghan, George Matich

**Affiliations:** ^1^Neuromorphic Sensor Signal Processing Lab, Centre for Image and Signal Processing, Electrical and Electronic Engineering, University of Strathclyde, Glasgow, United Kingdom; ^2^Leonardo MW Ltd., London, United Kingdom

**Keywords:** spiking, convolution, segmentation, tracking, STDP, neuromorphic, neural network, asynchronous

## Abstract

Traditionally the Perception Action cycle is the first stage of building an autonomous robotic system and a practical way to implement a low latency reactive system within a low Size, Weight and Power (SWaP) package. However, within complex scenarios, this method can lack contextual understanding about the scene, such as object recognition-based tracking or system attention. Object detection, identification and tracking along with semantic segmentation and attention are all modern computer vision tasks in which Convolutional Neural Networks (CNN) have shown significant success, although such networks often have a large computational overhead and power requirements, which are not ideal in smaller robotics tasks. Furthermore, cloud computing and massively parallel processing like in Graphic Processing Units (GPUs) are outside the specification of many tasks due to their respective latency and SWaP constraints. In response to this, Spiking Convolutional Neural Networks (SCNNs) look to provide the feature extraction benefits of CNNs, while maintaining low latency and power overhead thanks to their asynchronous spiking event-based processing. A novel Neuromorphic Perception Understanding Action (PUA) system is presented, that aims to combine the feature extraction benefits of CNNs with low latency processing of SCNNs. The PUA utilizes a Neuromorphic Vision Sensor for Perception that facilitates asynchronous processing within a Spiking fully Convolutional Neural Network (SpikeCNN) to provide semantic segmentation and Understanding of the scene. The output is fed to a spiking control system providing Actions. With this approach, the aim is to bring features of deep learning into the lower levels of autonomous robotics, while maintaining a biologically plausible STDP rule throughout the learned encoding part of the network. The network will be shown to provide a more robust and predictable management of spiking activity with an improved thresholding response. The reported experiments show that this system can deliver robust results of over 96 and 81% for accuracy and Intersection over Union, ensuring such a system can be successfully used within object recognition, classification and tracking problem. This demonstrates that the attention of the system can be tracked accurately, while the asynchronous processing means the controller can give precise track updates with minimal latency.

## 1. Introduction

Understanding and reasoning is a fundamental process in most biological perception action cycles. It is through understanding of our visual perception that helps to inform our basic decision-making processes like ‘friend or foe” and “edible or inedible,” which ultimately is key to progression or survival. Adding some level of understanding into this cycle can help to deliver a robust robotic system that could perform more complex variations of simple following and tracking tasks. Computer Vision (CV) has made this understanding a reality for robotics systems, with traditional CV methods providing simple feature extraction at low latency, or modern deep learning-based Convolutional Neural Networks (CNN) providing state of the art results in almost every task with high precision and accuracy, but at the cost of higher latency and computation throughput. This often leaves the CNN out of the reach of the small robotic system world due to its lower power and computational specifications. Modern research looks toward biological inspirations to help solve these tasks, by bringing forward neuromorphic robotics, which seeks to merge the computational advantages of system, such as the neuromorphic event-based vision sensor (NVS) and neuromorphic processors together, combined with Spiking Neural Network (SNN) which can allow for processing and control system structures. Typically a robotic system in this domain might aim to reach a Perception, Cognition, Action cycle, while the simpler approach of Understanding as a step toward cognition could be realized in an easier way, using the Perception Understanding Action (PUA) cycle as a stepping stone toward this goal.

Perception using neuromorphic vision sensors has become a promising solution. An NVS, as for example the Dynamic Vision Sensor (DVS) (Lichtsteiner et al., 2008), mimics the biological retina to generate spikes in the order of microseconds, in response to the pixel-level changes of brightness caused by motion. NVSs offer significant advantages over standard frame-based cameras, with no motion blur, a high dynamic range, and latency in the order of microseconds (Gehrig et al., 2018). Hence, the NVS is suitable for working under poor light conditions and on high-speed mobile platforms. There has been considerable research detailing the advantages of using an NVS in various vision tasks, such as high-speed target tracking (Lagorce et al., 2015; Mueggler et al., 2017) and object recognition (Kheradpisheh et al., 2018). Moreover, due to the fact that a pixel of an NVS is a silicon retinal neuron represented by an asynchronously generated spiking impulse, this can be directly fed into Spiking Neural Networks (SNNs) as input spikes for implementing target detecting and tracking in a faster and more neuromorphic approach.

Understanding through asynchronous spiking event-based computations like SNNs, often seen as the low latency biologically inspired alternative to CNNs, could provide an alternative solution to tracking and segmentation problems, through the ability to only compute on the currently active parts of the network, which in comparison to Artificial Neural Networks (ANN) and CNNs can require orders of magnitude less power consumption (Park et al., 2014). SNNs differ from normal computation processing and take inspiration from closer to biology, where expensive memory access operations are negated due to computations and memory being exclusively local (Paugam-Moisy and Bohte, 2012). Instead of using numerical representations like traditional methods, SNNs use spikes to transmit information with a key emphasis on the timing of those spikes. Several methods exist to train SNNs, with recent implementations seeing a conversion from CNN to SNN (Cao et al., 2015; Hunsberger and Eliasmith, 2015; Kim et al., 2019; Sengupta et al., 2019) yield promising results and open SNN architectures to the wider Machine and Deep Learning (ML-DL) audience. However, this method is still burdened with the training computational overhead and does little to utilize the efficiency of event driven computations. The SNN's Spike Time Dependent Plasticity (STDP) and spike-based back-propagation learning have been demonstrated to capture hierarchical features in SpikeCNNs (Masquelier and Thorpe, 2007; O'Connor et al., 2013; Panda et al., 2017; Kheradpisheh et al., 2018; Masquelier and Kheradpisheh, 2018; Falez et al., 2019). Both of these methods better equip the network to deal with event driven sensors, where the significant gains over CNNs could be realized.

This work aims to build on the already successful perception-action models (Nishiwaki et al., 2003; Xie, 2003; Bohg et al., 2017; Masuta et al., 2017) and add some semantic understanding to the robotic system. With image segmentation seen as a critical low-level visual routine for robot perception, a semantic understanding of the scene can play an important role for robots to understand the context in their operational environment. This context can then lead to a change in the action that could be undertaken. In this article, we show how using a spiking fully convolution neural network for event-based segmentation of a neuromorphic vision sensor can lead to accurate perception and tracking capabilities with low latency and computation overhead. Leveraging this spiking event-based segmentation framework to feed a spiking control system allows the low latency to continue from the perception to the action.

The PUA system presented builds on SpikeSEG, a spiking segmentation network from previous work (Kirkland et al., 2020), and extends it with a systematic approach to spike-based object recognition with tracking, lateral inhibition classifications, a new thresholding mechanism and modification to STDP learning process. Moreover, differently from Kirkland et al. (2020), the novel work presented is applied to a different application context, i.e., object recognition with attention. In light of this the novel contributions of this work include:

- SpikeSEGs segmentation output is integrated into a spike-based control system to produce the Perception-Understanding-Action system where the segmentation infers the attention of the system to allow controller track updates.
- The revised network includes more features to enhance the segmentation ability, including:
  – Lateral inhibition pseudo classification mechanism for semantic segmentation-based attention.
  – A new Pre-Empt then Adapt Thresholding (PEAT) approach designed to deal with potentially noisy, corrupt or adversarial inputs.
  – A modification to the STDP learning rules to include feature pruning (resetting) if under/over utilized.

The rest of the paper is organized as follows. Section 2 reviews related research topics covering each of the PUA framework individual sections. Section 3 presents the methodology, with an insight to each of the proposed system components. The results are detailed in section 4 and section 5 provides the conclusion.

## 2. Related Work

The allure of low latency object recognition and localization has brought the attractive features of the NVS (mainly the DVS) to the forefront of research. Early low latency control examples, such as the Pencil Balancer (Conradt et al., 2009) and the Robotic Goalie (Delbruck and Lang, 2013), help to highlight the latency advantages that an NVS can provide. Exploiting the sparse and asynchronous output of the sensor allow successful applications to these low latency reactive tasks. However, both systems fall short of fully capitalizing on the event-driven asynchronous output, through a processing and control regime of similar nature.

The concept of exploiting the NVS low latency continues into object tracking. Low latency tracking relies upon robust feature detection, with geometric shapes being ideal features to detect. A number of methods have been implemented successfully, such as geometric constraints (Clady et al., 2015) along with advanced corner detection methods, as for example Harris (Vasco et al., 2016) and FAST (Mueggler et al., 2017). The use of more complex features, such as Gaussians, Gabors, and other hand crafted kernels (Lagorce et al., 2015) provides a pathway to modern Convolutional Neural Network feature extraction approaches (Li and Shi, 2019), that implement a correlation filter from the learned features of the CNN. This allows a multi-level approach whereby correlations of intermediate layers can also be performed to improve the inherent latency disadvantage of the CNN approach, albeit with an accuracy trade-off.

Spiking Neural Networks have seen success with NVS data used for object detection and classification (Bichler et al., 2012; Stromatias et al., 2017; Paulun et al., 2018). Recent work has implemented Spiking Convolutional Neural Networks (Kheradpisheh et al., 2018; Falez et al., 2019) with NVS-like data created using a difference of Gaussian filter, suggesting the combination of SNNs and Deep Learning could yield successful results (Tavanaei et al., 2019). SNNs have also been utilized for tracking with an NVS through implementations inspired by the Hough Transform (Wiesmann et al., 2012; Seifozzakerini et al., 2016; Jiang et al., 2019), to be able to detect and track lines and circles. Spiking Neural Networks can also be utilized to implement control systems, from simple altitude control (Levy, 2020) to an adaptive robotic arm controller (DeWolf et al., 2016). Ultimately the majority of research only utilizes one aspect of the SNN, either processing or control. Even though SNNs have been shown to implement a full perception cognition action cycle with Spaun (Eliasmith et al., 2012), underpinning the ideology of a fully spike-based neuromorphic system similar to that proposed with the Perception Understanding Action framework in this paper.

## 3. Methodology

### 3.1. Perception-Understanding-Action Framework

**Figure 1:**
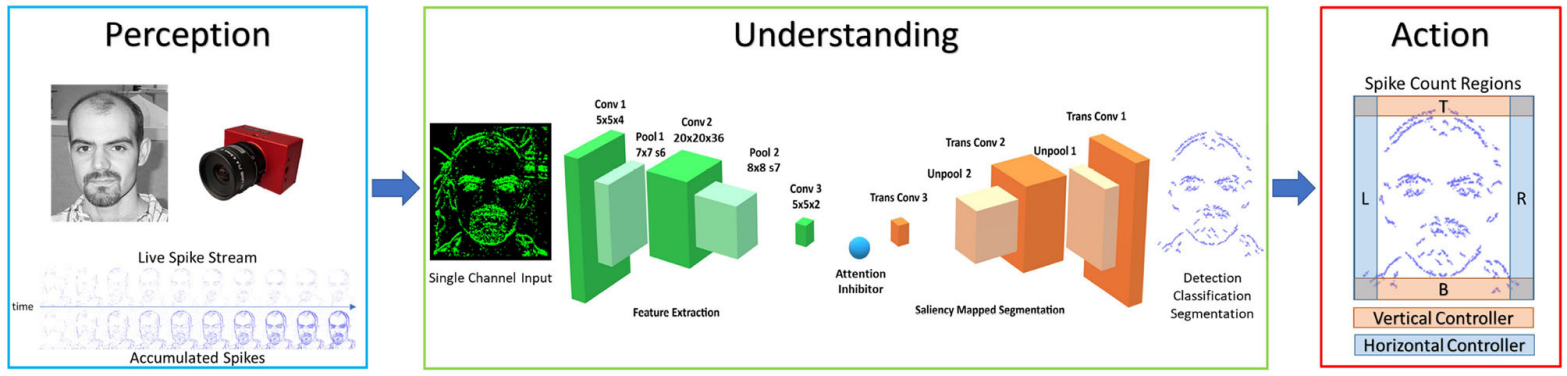
Perception understanding action framework, with internal system diagrams showing the perception input [image from Caltech Dataset (Li et al., 2004), the Understanding network SpikeSEG (Kirkland et al., 2020), and the Action controller method].

The Perception-Understanding-Action framework specifies how the system will utilize the asynchronous event driven nature of the Neuromorphic spiking domain, and it is illustrated in Figure 1. In the Perception block, the NVS is used to sparsely and asynchronously encode the luminosity changes within the scene. In the Understanding block, inputs are understood through the use of the Encoder-Decoder SpikeCNN [SpikeSEG (Kirkland et al., 2020)] contextualizing and building understanding of the scene through semantic segmentation. In the Action block, the segmented output is used to provide an input to the spike counters at the edge of the field of view, allowing a simplistic semantic tracking controller to be realized. This control output would then be able to influence motors or actuators to allow an asynchronous end to end Neuromorphic system. This system aims to provide a low latency competitor to the Perception Action robotic system where the sensor input is directly fed to the controller, while providing an upgraded feature representation to the more complex line and edge detection-based approaches. The system can even provide benefits or replace some computer vision-based robotic tasks which utilize CNNs for complex feature extraction, while providing lower latency and computational overhead. Furthermore, compared to the CNN, the SCNN provides a more readily understandable processing stage, where features are sparse and more visually interpretable.

### 3.2. Perception

A key element in producing a low latency system with a low computational overhead is to have a sensor that can exploit the sparse and asynchronous computational elements of an SNN while still giving a detailed recording of the scene. Neuromorphic Vision Sensors (NVS) *(event-based Vision Sensors*) (Lichtsteiner et al., 2008; Brandli et al., 2014) have recently become more popular and widespread. These camera-like devices are bio-inspired vision sensors that attempt to emulate the functioning of biological retinas. They differ from conventional cameras in that, they don't record all the information the sensor sees at set intervals. Instead these sensors produce an output only when a change is detected. This in turn means they are capturing the luminosity at a set point in time, meaning a continuous temporal derivative of luminosity is output. Whenever this happens, an event *e* = [*x, y, ts, p*] is created, indicating the *x* and *y* position along with the time *ts* at which the change has been detected and its polarity, where *p* ∈ {1, −1} is a positive or negative change in brightness. This change in operation not only increases the sparsity of the signal but allows for it to output asynchronously. Resulting in microsecond temporal resolution and considerably lower power consumption and bandwidth. These parameters make the NVS an ideal candidate for object tracking, especially of fast moving objects (Delbruck and Lichtsteiner, 2007; Glover and Bartolozzi, 2017), however many methods are still yet to utilize this spiking sensor within a match spiking processing, such as SNNs.

### 3.3. Understanding Through Spiking Segmentation

The Understanding of this system is inferred from the semantic segmentation operation carried out by the SpikeSEG network (Kirkland et al., 2020), seen in Figure 1 within the Understanding block. The SpikeSEG segmentation network has received a number of improvements and upgrades along with its integration within the PUA framework.

#### 3.3.1. Network Architecture

The network architecture illustrated within Figure 1 (Understanding) is made up of two main sections seen in green and orange, that relate to the encoding and decoding layers, respectively. The network is split into these two sections where training only occurs on the encoding side, while the weights are tied to the mirrored decoding layers. This allows a integrate and fire neuron with layer-wise STDP mechanism with adaptive thresholding and pruning to be used to help compress the representation of the input to allow the decoding layer to segment the image based on the middle pseudo classification layers. This encoding-decoding structure symbolizes a feature extraction then shape generation process. The learning of the encoding process aims to extract common spatial structures as useful features, then decode those learned features over to the shape generation process, unraveling the latent space classification representation but with a reduction in spike due to the max pooling process. The network has nine computational layers *(Conv1-Pool1-Conv2-Pool2-Conv3-TransConv3-UnPool2-TransConv2-UnPool1-TransConv1)* as seen in Figure 1. Between the Conv3 and TransConv3 layers, there is a user-defined attention inhibition mechanism, which can operate in two manners: No Inhibition, which allows semantic segmentation of all recognized classes from the pseudo classification layer; or With Inhibition, that only allows one class to propagate forward to the decoding layers. This attention not only provides a reduction in the amount of computation, but also simplifies the input to the controller.

#### 3.3.2. Encoding

**Figure 2:**
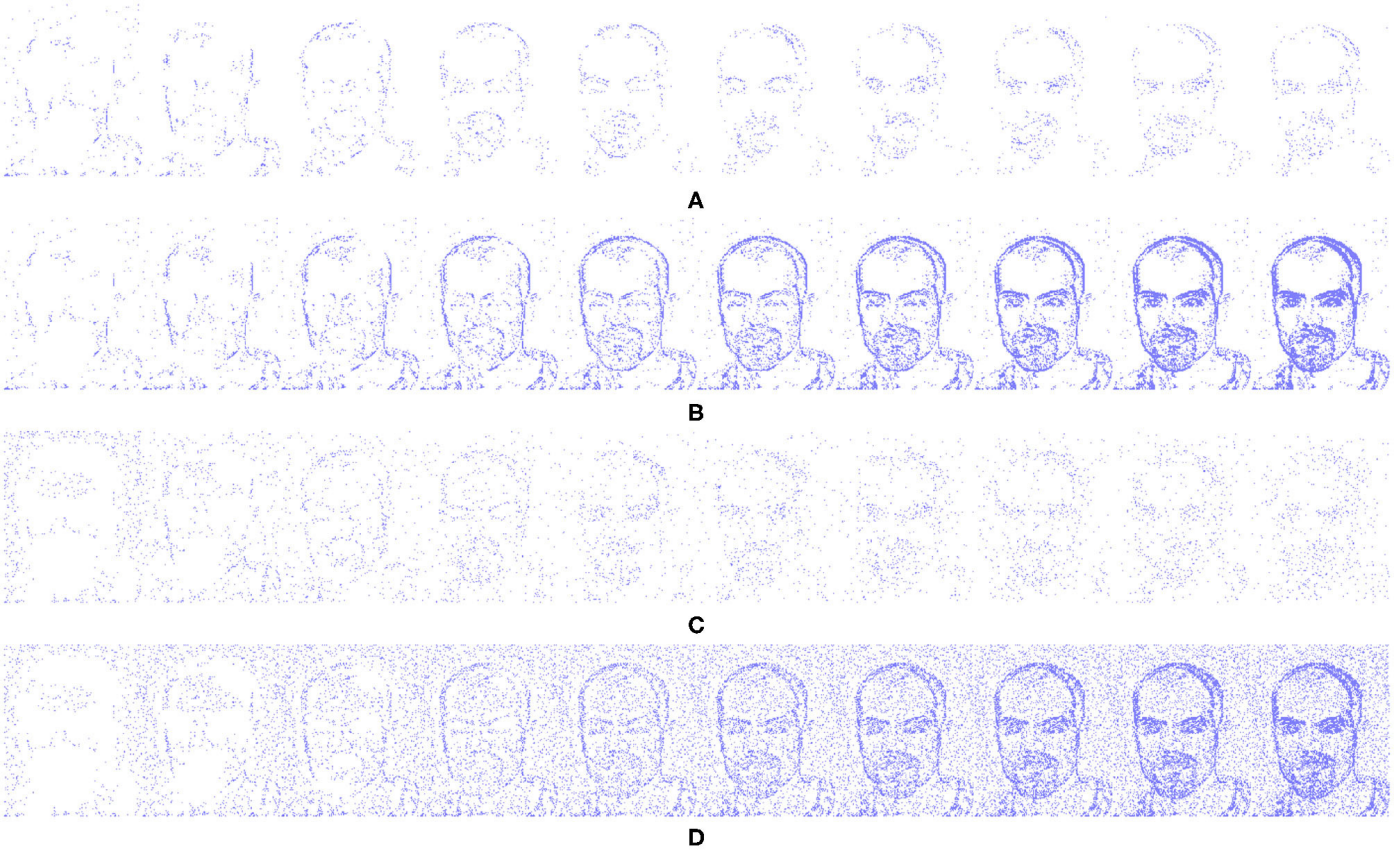
Input event streams from N-Caltech Dataset “Face,” with **(A,B)** showing a 10 ms clip over 10 steps going from left to right. **(A)** Showing the input to the network per step and **(B)** showing the accumulated inputs for easier visualization. **(C,D)** Show a 10 ms clip over 10 steps with additive noise to show how extra noise affects the input stream, with **(C)** showing per step and **(D)** showing accumulated.

The encoding part of this system is derived from a basic SpikeCNN with a simplified STDP learning mechanism (Kheradpisheh et al., 2018). To allow the network to better suit the framework and encoding decoding structure a number of modification are applied. As the structure of the network is now fully convolutional there is no longer a requirement for a global pooling layer for classification. Instead the final convolution layer is utilized as a mock classifier by mapping the number of known classes to the number of kernel used for feature learning. This method is also used to help the interperitability of the system as having one kernel per classes allows for better visualization of the network features. Through the use of a modified STDP rule and adaptive neuron thresholding, the encoder aims to capture the reoccurring features that are most salient through the event stream inputs. The input events are fed into the network via a temporal buffering stage, to allow for a more plausible current computing solution, such as on the Intel Loihi Neuromorphic chip (Davies et al., 2018), while ideally they would just be a constant stream. To internally mimic the continuous data, 10 ms of event data is buffered into 10 steps, representing 1 ms each (this value of 10 ms is chosen to empirical testing and based on the input spike count of the N-Caltech Dataset); this input data stream is shown in Figure 2. Figure 2, also illustrates what 1 ms of data looks like over the 10 ms (A) and how it looks if accumulated over 10 ms (B). Figure 2 then demonstrates how added noise affects the input stream, repeating the images in Figures 2A,B with noise in 1 ms steps in (C) and accumulated over 10 ms in (D). For each time step in the encoding processing, a spike activity map *Sk*_*mt*_ is also produced, where *m* is the feature map and *t* is the time step. This allows an account of the exact spatial time location of each active pixel used in the encoding processing, which helps allow the decoder to map these active areas back into the pixel space.

#### 3.3.3. Decoding

**Figure 3:**
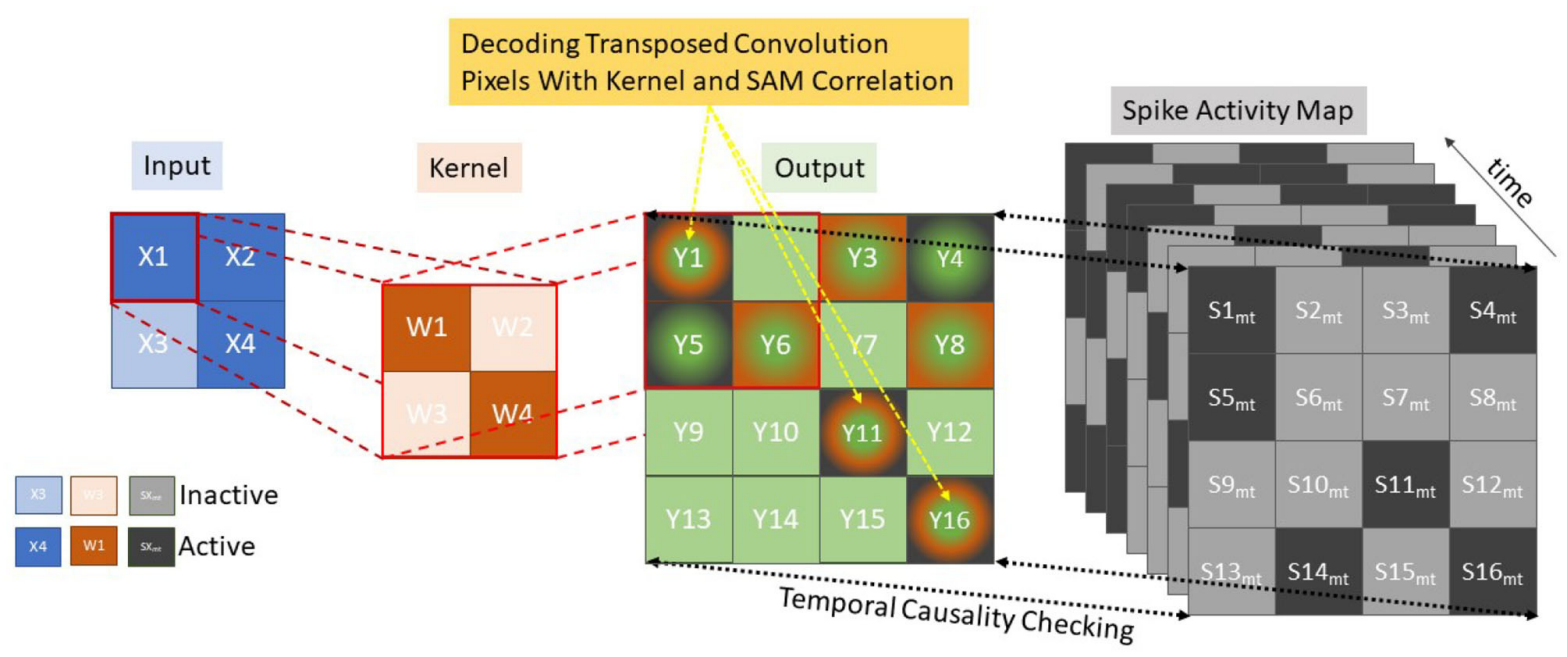
Decoding using transposed convolutions with spike activity mapping, resulting in active pixel saliency mapping.

The Decoding Process makes use of the same unpooling and transpose convolutions as (Simonyan et al., 2013; Zeiler and Fergus, 2014; Long et al., 2015; Badrinarayanan et al., 2017) taking pixels in the latent classification space back into the original pixel space. However, no learning mechanism is used, as the mapping is based on temporal activity and pixel saliency mapping, utilizing a similar method to tied weights (Hinton et al., 2006) and switches (activations within the pooling layers) from the encoding layer to map directly to the decoding such that *W*_*ij*(*encoding*)_ = *W*_*ji*(*decoding*)_. This modification is required to deal with the temporal component of the spiking network, as now the latent pixel space representation must be unraveled with the constraints and context of space and time. Changes are made to both the transposed convolutions and the unpooling layers. The transposed convolution still functions as a fractionally strided convolution of the weight kernel as normal. However, now an extra step of comparing the output mapping with a temporal spike activity map of the post-convolution pixel space is required as illustrated in Figure 3, where the conventional Input via Kernel to Output stage remains, with an added Spike Activity Map check on each term in the output for temporal causality.

Since the encoding neurons emit at most one spike per buffered time input, the Spike Activity Map is used to keep track of the first spike times (in time-step scale) of the neurons. Every stimulus is represented by M feature maps, each constitutes a grid of neurons seen as a kernel value K, equal to the row-major linear indexing of the kernel. Let *Tp* be the processing steps between the tied encoding and decoding layer with a maximum possible difference of nine processing time-steps (five encoding and decoding layers each). While each encoding layer has a value *Te*_*m,k*_, which denotes the spike time of the encoding neuron placed at position (k) of the feature map m, where 0 ≤ *m* < *M*, 0 ≤ *k* < *K*. The individual decoding layer then considers this stimulus as a three-dimensional binary spike tensor S of size *Tp*_*max*_ × *M* × *K* where a spike in the decoding layer *Sd* is a function of:

(1) $$\begin{array}{l} Sd\left(Tp,Te,m,k\right)=\{\begin{array}{cc} 1 & Td_{m,k}=Te_{m,k}+Tp\\ 0 & otherwise \end{array} \end{array}$$

Where the decoding time *Td*_*m,k*_ for each map and kernel value is compared to the equivalent encoding layer *Te*_*m,k*_ offset by the processing time *Tp*. It is this *Te*_*m,k*_ + *Tp* that is represented by the Spike Activity Map shown in Figure 3 where *Sk*_*m,t*_ is illustrated as the process ensuring *Td*_*m,k*_ = *Te*_*m,k*_ + *Tp* while “Output” demonstrates an example of the transposed convolution process. To reduce memory overhead only the last 9 Spike Activity Maps as this is the minimum requirement to ensure temporal causality. Within Figure 3, the green and orange squares represent the transposed convolution outputs and the green, orange and black outputs represent the outputs from the transposed convolution decoding that also matched up with encoding layer, through correlation with the Spike Activity Map. This demonstrates how the Spike Activity Map reduces the “Output” values to only those with equivalent temporal values. The saliency mapping occurs within the unpooling layers which operate on a similar manner in order to keep temporal causality, but due to the max pooling operation working in reverse only one pixel per pooling kernel is processed. With reference to Figure 3, this would mean the orange kernel would only have one active square, which reduces the output significantly. The measure allows only the most salient features to propagate through the decoding layers, resulting in the segmentation with only those features that best fit the pseudo classification. A verbal illustration being, if there are nine time steps between Conv-1 and TConv-1, while only five steps between Conv-2 and TConv-2 and one step between Conv-3 and TConv-3. So, if a spike occurs at time step 2 within Conv-1, the temporal check will only allow TConv-1 to allow a spike at that location at time step 11.

#### 3.3.4. Adaptive Neuron Thresholding

The adaptive neuron thresholding used within this paper builds upon the Pre-Emptive Neuron Thresholding (Kirkland et al., 2019, 2020). Improvements are made by no longer solely relying on synaptic scaling from the input number of spikes as a means of homoeostasis. Although this was successful in stopping the progression of less structured noise features within the first convolution layer and structured noise when synaptic scaling was applied to all layers. Along with the structured noise filtering process, this homoeostasis rule also accidentally removes some of the less common desired features from propagating as discrimination between these and noise from input spike count is insufficient. The update to the algorithm sees an adaptive element in the form of intrinsic layer-wise synaptic scaling (a layer-wise spike counter) added to the thresholding parameter to potentially counter this less common feature removal. During training the thresholding is set as follows

(2) $$\begin{array}{l} V_{thr}(S_{in},S_{l})=\{\begin{array}{cc} \frac{K_{l}}{4} & \text{for }S_{in}<S_{in(min)}\\ \begin{array}{cc} c+mV_{thr}+h^{-} & \text{for }S_{l}<H_{l}\\ c+mV_{thr}+h^{0} & \text{for }S_{l}=H_{l}\\ c+mV_{thr}+h^{+} & \text{for }S_{l}{\text{=H}}_{l} \end{array}\} & \text{for }S_{in(min)}<S_{in}<S_{in(max)}\\ \frac{K_{l}}{2} & \text{for }S_{in}>S_{in(max)} \end{array} \end{array}$$

Where *V*_*thr*_ is the neuron threshold, dependent on both the spiking input rate, *S*_*in*_, and the layer-wise spike rate, *S*_*l*_. *m* is gradient of the linear relationship between *V*_*thr*_ and *S*_*in*_, with *c* being the y-intercept. *h* the homoeostasis offset is determined to be either positive, negative or zero dependent on the layer-wise spike count, *S*_*l*_ when compared to the set homoeostasis value *H*_*l*_. While *K*_*l*_ is the convolution kernel size within that layer. The equation follows a piecewise function such that *V*_*thr*_ is described as $\left\{V_{thr}\in \mathbb{N}|\frac{K_{l}}{4}<V_{thr}<\frac{K_{l}}{2}\right\}$. When the spike input rate *S*_*in*_ is within a normal range, the function is then defined by the bounded linear relationship with the homoeostasis offset. The values of *h*^−^, *h*^0^, *h*^+^ and *H*_*l*_ are set through empirical testing by monitoring the range of *S*_*l*_ and *S*_*in*_ values from the N-Caltech dataset.

Once training is complete and the features within the convolution kernels are known, the thresholding changes to take into account the size of the feature, as the range of threshold values might now be smaller than in the training stage. This modification changes the outer bounds of the threshold as shown

(3) $$\begin{array}{l} V_{thr}(S_{in},S_{l})=\{\begin{array}{cc} \frac{F_{min}}{2} & \text{for }S_{in}<S_{in(min)}\\ \begin{array}{cc} c+mV_{thr}+h^{-} & \text{for }S_{l}<H_{l}\\ c+mV_{thr}+h & \text{for }S_{l}=H_{l}\\ c+mV_{thr}+h^{+} & \text{for }S_{l}=H_{l} \end{array}\} & \text{for }S_{in(min)}<S_{in}<S_{in(max)}\\ F_{min} & \text{for }S_{in}>S_{in(max)} \end{array} \end{array}$$

Where *F*_*min*_ is the smallest feature size within that layer. This parameter change ensure the threshold value does not exceed the smallest feature size, which would result in that neuron being unable to reach firing potential. In both cases the training and testing the input spike count *S*_*in*_ value affects the threshold for each input spike buffer, while the layer-wise spike count *S*_*l*_ is average over 10 inputs.

This allows a layer-wise adaptability dependent on the amount of spiking within the previous layer. The algorithm now permits a high volume of spiking activity at the input to be initially pre-emptively dealt with, ensuring a large amount of spiking activity does not reach the controller, causing an undesired response. Then adapting the thresholds to allow sufficient spiking activity ensures a smoother and more robust controller output of the system. The key element of this method is to ensure a more robust and predictable outcome when a noisy, corrupt or adversarial input is received. With this being more of a concern due to the system be asynchronous end to end, a high volume incoherent input could directly lead to a wild or undesired response from the controller. This approach errs on the side of caution with the sudden increase in input spikes being inhibited first, and then excited to a desired level, in contrast to a typical intrinsic response of allowing the activity, and then inhibiting to a desired response.

#### 3.3.5. Changes to STDP Training With Active Pruning

A simplified unsupervised STDP rule (Bi and Poo, 1998; Kheradpisheh et al., 2018) is used throughout the training process, including a Winner Take All (WTA) approach to STDP, that operates by only allowing one neuron (feature) in a neuronal map (feature map) to fire per time constant; this is viewed as an intra map competition. This WTA approach then moves onto the inter map inhibition, only allowing one spike to occur in any given spatial region, typically the size of the convolution kernel, throughout all the maps. As a result of these inhibition measures, two features can tend toward representing the same feature until such point where one becomes more active, while the other gets inhibited to the point of infrequent or no use. At this stage the feature representation has become obsolete and can be pruned or reset, allowing the opportunity to form another more useful feature. To capture this information the layer-wise training method make use of the training layers convergence values

(4) $$\begin{array}{l} C_{l}=\underset{k}{\sum }\underset{i}{\sum }\frac{w_{ki}\left(1-w_{ki}\right)}{n_{w_{ki}}} \end{array}$$

Where *C*_*l*_ is the convergence score for the layer and *w*_*ki*_ is the *i*th synaptic weight of the *k*th convolution kernel. The *n*_*w*_*ki*__ is the number of individual weights contained with the layer calculated by kernel size and the number of kernels in the previous and current layers, *n*_*w*_*ki*__ = *K* × *k*_*pre*_ × *k*_*cur*_. The pruning function makes use of the convergence score that is typically used to indicate when training is complete, as the convergence tends to zero due to the weights tending to 0 or 1. Noticing that the layer-wise convergence is just a sum across all the kernels allows a modification to calculate the convergence across each kernel within that layer with respect to all previous maps.

(5) $$\begin{array}{l} C_{k_{cur}}=\underset{k_{pre}}{\sum }\underset{i}{\sum }\frac{w_{k_{pre}i}\left(1-w_{k_{pre}i}\right)}{n_{w_{k_{pre}i}}} \end{array}$$

This new terms *C*_*k*_*cur*__ allows monitoring of each kernel during the learning process, as previously mentioned obsolete kernels that learned similar features are less active, resulting in higher convergence numbers while maintaining a high spiking activity. The high spiking activity is due to the kernel maintaining the high starting weight value which are random values drawn from a normal distribution with the mean of μ = 0.8 and standard deviation of σ = 0.05. However, the kernel does not exhibit a feature that allows it to spike quick enough to receive a weight update from the STDP WTA rule. As the kernel had already started a convergence to a particular feature, once under-active it then attempts to convergence to another commonly occurring feature. However, the kernel often convergences to a useless feature representation that is unhelpful to the final result of the network. This pruning method, rather than simply removing the kernel, gives it the chance to learn a new feature from scratch by resetting the kernels weights. Thus, allowing the best chance of convergence to a useful feature. This pruning process takes place once the convergence value of the layer *C*_*l*_ drops below the original starting value. As initially the weights are deconverging from the mean weight initialization, before returning to the original convergence value on the way to zero. Once this milestone has been reached the pruning function in activated

(6) $$\begin{array}{l} Prune_{k_{cur}}\left(C_{k_{cur}},C_{l},S_{k}\right)=\{\begin{array}{cc} 1 & \text{for }C_{k_{cur}}>\overset{-}{C_{l}}+1\sigma_{C_{l}}\text{ and }S_{k}>\overset{-}{S_{l}}+3\sigma_{S_{l}}\\ 0 & \text{otherwise} \end{array} \end{array}$$

where $\overset{-}{C_{l}}$ is the mean convergence for that layer, σ_*C*_*l*__ is the standard deviation of that layers values, *S*_*k*_ is the spike activity within an individual kernel. $\overset{-}{S_{l}}$ is the mean spike count of that layer and σ_*S*_*l*__ is it standard deviation. If a kernel value has a convergence score higher than 1 STD from the mean while having a spiking activity 3 STD higher than the mean spike rate in that layer, the kernel is reset with the initial weight distribution. Since many of the kernels are already converging to useful features this newly reset kernel will convergence to a new unrepresented feature.

#### 3.3.6. Latent Space Inhibition for Attention

In order to have the network change its focus or attention, the latent space pseudo classification layer also acts as an inhibition layer for this mechanism. This operates by inhibiting other neurons in that layer if a specific neurons feature is chosen to be the attention. This is an external mechanism to the network as otherwise, the network will give equal attention to the full scene and semantically segments all known objects within a scene. This allows a simplification of the output of the network fed to the controller, allowing the attention of the system to be narrowed to that particular pseudo-class. This segmentation-based attention can then be used to follow a given class dependent on the output of the controller. It operates between convolution layer 3 and trans-convolution layer 3 with the same principals as the inter map inhibition with the encoder, though now the spatial region is the whole latent space. This inhibition can also work autonomously where the pseudo-class with the most activity is the attention of the network, allowing the network to switch attention to known classes based on their prevalence within the scene.

### 3.4. Tracking With Attention

The Action part of the system with its spiking controller is directly influenced by the attention mechanism, as when no attention is chosen the controller acts on all the segmented data being output by the SpikeSEG network. This could cause unwanted control output if the scene contained more than one known class, as unknown classes should still be removed by the process. Once a class has been chosen as the attention, the segmentation output is reduced to only that class, as illustrated in Figure 1 (Action), which allows for simple spike counter controller to produce a more robust and reliable output. The reduction in information initially by the NVS which then further reduces through the semantic segmentation and attention, allow the implementation of this simple spike counter. This is due to the segmentation output only containing information relating to the attention of the network, the controllers task is just to keep this in the center of the field of view. The simplicity of the controller also allows it to take advantage of the asynchronous event-driven system to provide low latency tracking updates a key element of the system. However, if there was more than one instance of a class in a scene there is no way to separate the two instances, so tracking would be based off all instances of a class. Nevertheless, this system would make an improvement over the purely spiking activity tracking systems by adding some semantic context to the activity, while the simplified spike counter in this instance allows class based tracking could be enhanced with more complex spike tracking, such as dynamic neural fields (Renner et al., 2019).

## 4. Results

In this section, a series of experiments on individual and multi event-stream recordings are presented. The metric used in this paper is the Intersection over Union (IoU, also known as the Jaccard Index) to grade the segmentation, which guides the control system of the network and ultimately, with user choice, the Attention of the system. This metric was used due to the availability of the bounding box annotations within the subset of the N-Caltech dataset that was used within the experiments. The feasibility of the attention-based tracking is also encapsulated within the IoU value, though due to the small saccade movements of the camera within the N-Caltech dataset, it is infeasible to use this to highlight spike-based tracking. This is due to two issues throughout the movements. The first is the IoU value only receives a small change as the displacement is often <10 pixels. The second is that the occurrence of segmented spike activity in the controlled regions, is due to the tight field of view around the class in scene. This results in the testing of the Perception and Understanding system only with this data. To ensure testing of the full Perception, Understanding, and Action system, two further experiments were carried out. The first with multi input streams on a large input space and the second using our own captured DVS data of a desk ornament with a hand held sensor. Lastly, the results sections show how the system is more robust and interpretable than alternative models, with the use of the Pre-Empt and Adapt Thresholding and the contour like sparse features within the weights of SpikeSEG.

Within these experiments the step time for any processing is now linked to the input time step, meaning internal propagation of spikes takes one step (or 1 ms) per layer, resulting in a 11 ms lag to get the segmented results. This allows for better visualization of the asynchronous manner of the processing and control for each step. However, this does not reflect the actual processing time of the network which, given its complexity compared to similar models ran on neuromorphic hardware, would most likely be able to execute this task in real-time for the 1 ms step, meaning a full pass through the network per input step. However, testing in this manner would not fully highlight the asynchronous advantage especially within a dynamic environment.

One further note is that throughout all the testing the features of Convolution Layer 1 are pre-set to best found features for initial edge detection, which results in a horizontal, vertical and two diagonal lines which can be see later in the Interpretablity section 4.3.2 within **Figure 14**.

### 4.1. Perception to Understanding With Segmentation

**Figure 4:**
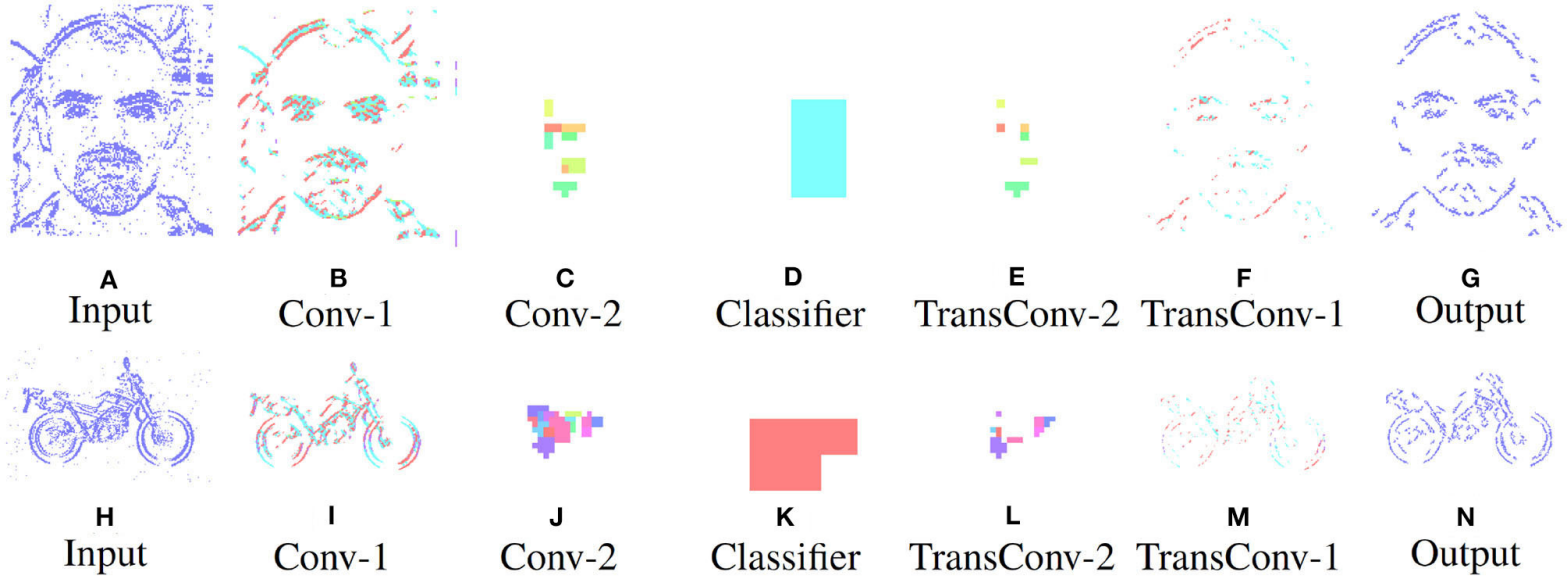
Segmentation performance of the network on an example face **(A–G)** and motorbike **(H–N)**, highlighting the encoding transition into the latent space used for pseudo classification **(A–D,H–K)**, then retracing of chosen features back to pixel level **(D–G,K–N)**.

**Figure 5:**
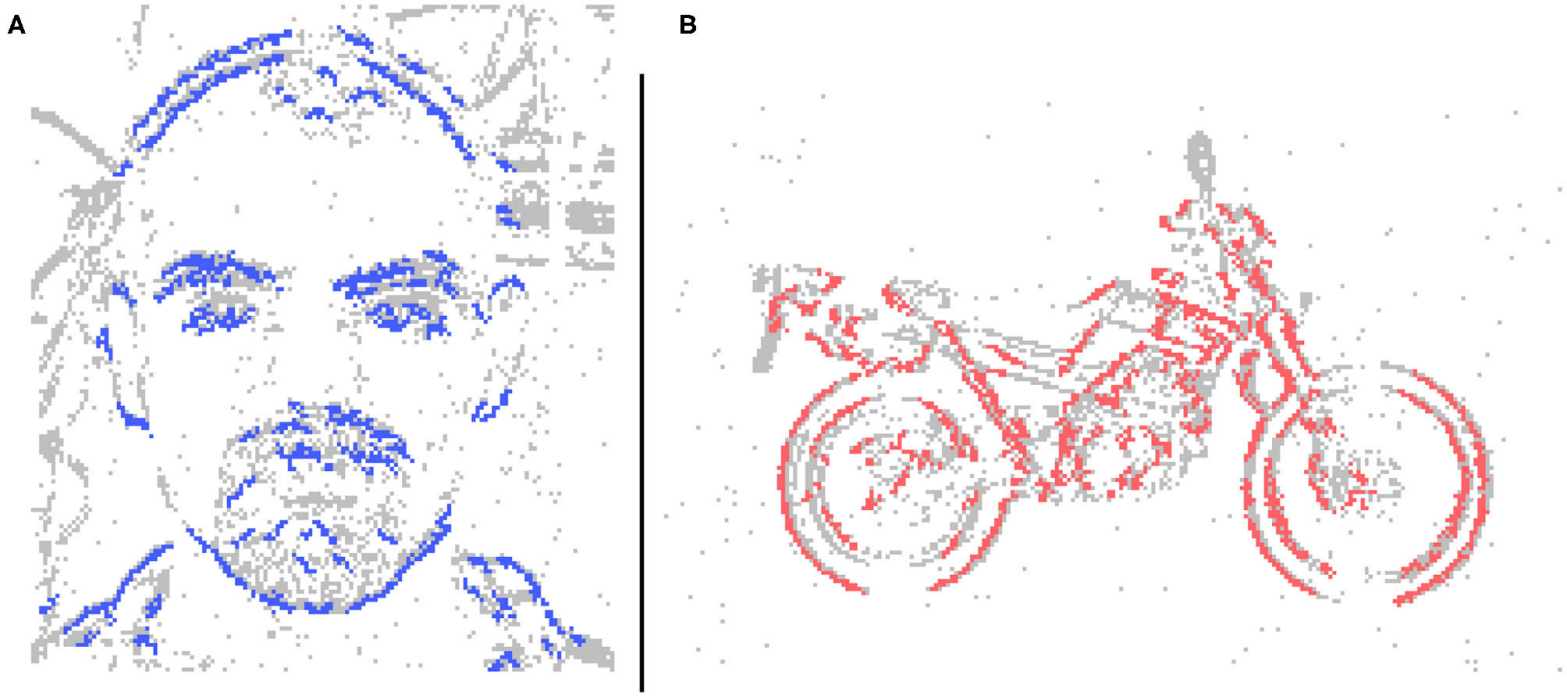
Segmentation overlays for the **(A)** Face and **(B)** Motorbike class from the N-CalTech dataset.

**Figure 6:**
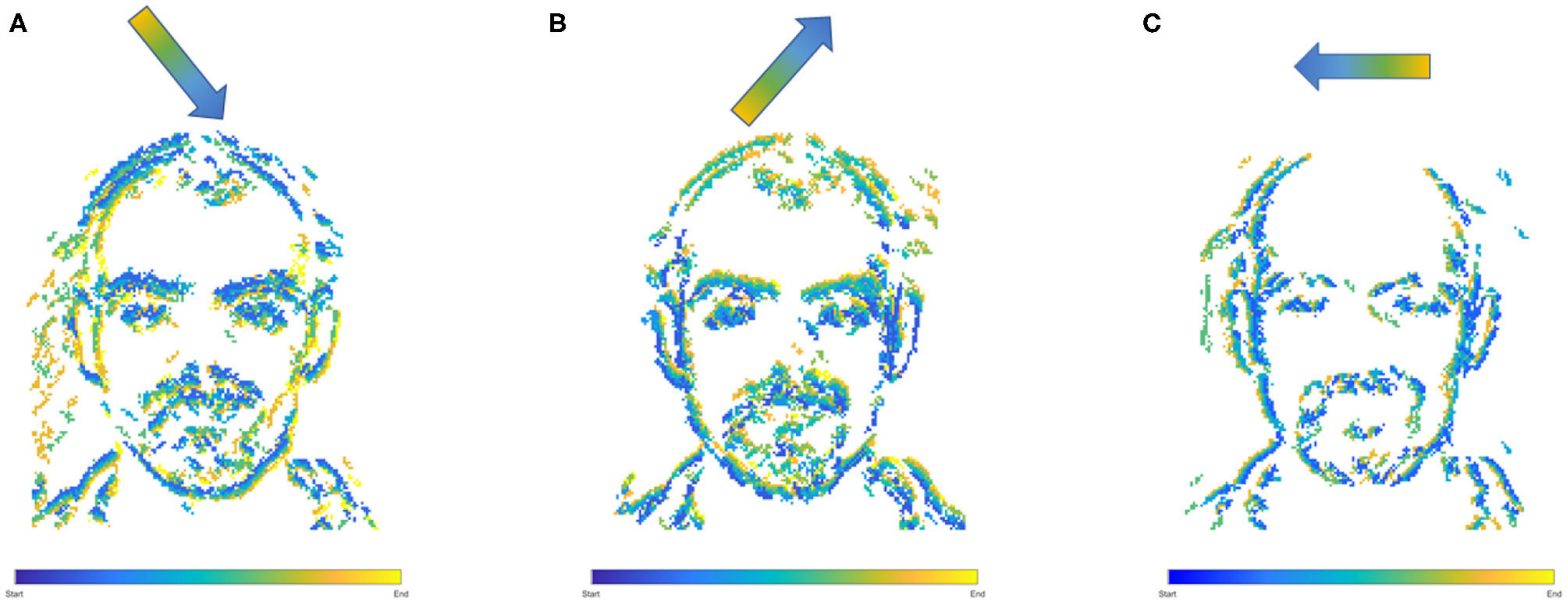
Overlapped Segmentation output over the complete event stream, showing the triangle of movements over the three saccades, **(A)** first movement, **(B)** second movement, **(C)** third movement.

Initially two subset classes from the N-Caltech dataset (Orchard et al., 2015) are used to evaluate the Understanding section of the system. On this single stream input typically only containing a singular class with variable amounts of background noise and clutter, the network is able to gain an accuracy of 96.8% within the pseudo classification layer and a 81% mean Intersection over Union score over each of the 10 ms buffered input that resulted in successful segmentation, results are also shown in Table 1. This is an improvement on the single results seen within (Kirkland et al., 2020) of 92 and 67% for accuracy and IoU, with the improved feature creation allowing a more detailed representation allowing an improvement in both the accuracy and segmentation. The test results are based on training with 200 samples from the Face and Motorbike classes with another 200 used for testing. This number was limited as the “Easy Faces” has just over 400 images and was converted into “Faces” within the N-Caltech dataset with the “Faces” category being removed. Four hundred images provided an equal training set between the Face and Motorbike classes. The images in Figure 4 shows how the segmentation process was completed firstly through encoding the event stream input through three convolution and two pooling layers (Figures 4B–D,I–K), resulting in a sparse latent space representation used to provide a classification of this binary task (Figures 4D,K). Figure 4, then shows how the classification locations are then mapped back onto the pixel space through the undoing of the three convolutions and two pooling layers (Figures 4E–G,L–N). For illustrative purposes, both the face and motorbike are accumulations of the network activity according to 10 ms input buffer and full propagation of spikes through the network. Each convolution process is shown, with pooling omitted, Convolution Layer 1 is shown in Figures 4B,I while layer 2 (Figures 4C,J,D,K) showing the third convolution also used as pseudo classification. Figures 4E,L show the second transposed convolutional layer, named to mirror the encoding side, while Figures 4F,M show transposed convolution 1 and Figures 4G,N display the segmented outputs. This segmentation result is shown overlapped onto the input for two examples within Figure 4. The colors used within Figure 5 are linked to the corresponding feature that was activated in that layer with Figures 4C,J showing different colored features active for each the face and motorbike, with section 4.3 exploring what the feature maps contain. This output from the SpikeSEG network can feed directly into the spiking controller of the PUA system, guiding any movement that would be required to follow the attention of the system. Although the controller in this context is unable to operate due to the narrow field of view and limited movement, the Understanding section of the system does still capture this small saccade movement of the camera within the segmented output as seen in this overlapped output image, Figure 6A showing a downward and right shift of the segmented pixels over time, relating to the inverse movement performed by the camera, while Figures 6B,C show the two further saccade movements. The segmentation also maintains an IoU value of above 0.7 throughout the movement, meaning the segmentation is of good quality throughout (0.5 being acceptable, 0.7 being good, and 0.9 being precise) (Zitnick and Dollár, 2014), for reference if the full input size is used for IoU the average output is ~0.57. Consequently, this means tracking would still be possible through alternative non-spiking methods such bounding boxes or centroid/center of mass calculation, but would remove the all spiking asynchronous feature of this system.

**Table 1:** Results from each of the experimental setup, listing both the accuracy and intersection over union.

**Dataset**	**Classification accuracy (%)**	**Intersection over union (%)**
N-CalTech (2 class)	96.8	81
N-CalTech (5 class)	86	76
N-CalTech (10 class)	75	71
Multistream N-CalTech	96.8	81
Multistream N-CalTech with noise	95.1	79
Panda	94	75

#### 4.1.1. N-Caltech Dataset Extended

**Figure 7:**
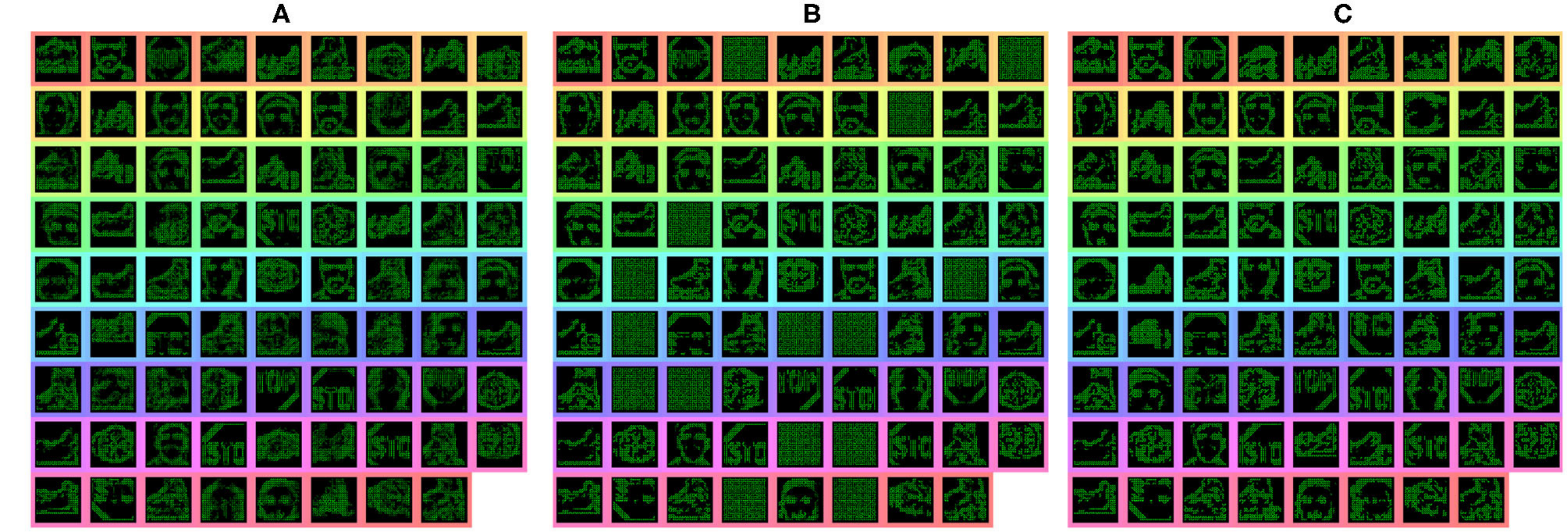
Features from the second convolution layer during training highlighting the pruning process. **(A)** Highlights the features prior to pruning, **(B)** shows which feature were reset to initial parameters, and **(C)** shows the newly learned features.

To further evaluate the scalability of the model, a further two experiments are carried out with 5 and 10 classes. This allowed testing the model with 2, 5, and 10 classes within the same experimental parameters, that being 16 features per class in second convolution layer and 1 per class in the third convolution layer, with active thresholding and pruning. Sixteen features was found to be a suitable value for number of features through prior empirical testing, where more features gave no further improvement, while less features was unable to capture the variation of some classes. The further classes added are: Inline Skate, Watch and Stop Sign for the 5 class, while Camera, Windsor Chair, Revolver, Stegosaurus and Cup are added for the 10 class experiment. These classes are chosen due to low variability in image spatial structure. As the network is only looking for natural spatial structural similarity avoidance of classes which have a large intraclass variance compared to the overall interclass relationship (Zamani and Jamzad, 2017). With this in mind and due to some the additional classes having a smaller number of sequences, the number of training and testing instances was changed to suit, at 20 training and 10 testing. Overall the network was able to achieve classification accuracies of 86 and 75% and IoU values of 76 and 71% for the 5 and 10 classes, respectively, results are shown in Table 1. The decrease in overall accuracy with additional classes is to be expected at the features built in the second convolution layer tend to get more similar. This is visually detailed in section 4.3.2 with the Interpretability showing the different features learned in the convolution layers. With this closer similarity of layer-wise features, an example of how the active pruning mechanism is shown in Figure 7, where a number of the features within the second convolution layer have a slower convergence rate while maintaining a high spike activity. This typically suggests the feature is not very discriminative and is an ideal candidate for being reset to learn a new feature. Figure 7, shows the original features just prior to reaching the pruning check point within (A), then indicates which features are chosen to prune with the feature being reset to random initialization within (B), the finally resulting in new features shown in (C).

Drawing insight from the result, within the 5 class experiment the inter class variance was high. However, once the 10 classes were added this inter and intra class variances seems to overlap. Resulting in many of the classes relying on similar features constructed from circles, with Motorbike, Cup, Camera, Watch, Stop Sign, and Face at times producing features are that undistinguishable from one another. It was also noted that as the number of classes increased the difference between average number of features in a kernel per class (that is ones that can be recognized as belonging to a particular class) leads to a higher likelihood that the class with the highest average feature number will be the most active. Within the last experiment with the 10 classes this was prevalent within the Revolver class as it had an average feature count in convolution layer 2 of around 200, while the average for camera was 110. This results in a higher chance that the revolver was classified by mistake ultimately bringing the overall accuracy down.

### 4.2. Perception, Understanding, and Action

This section is split into two parts both further testing the full PUA system, the first continues using the N-Caltech Dataset, however with multiple simultaneous inputs. The second part makes use of recorded data of desk ornament from a hand-held NVS to provide a further example of how the system works within another test environment and how the action part of the system deals with a moving class.

#### 4.2.1. N-Caltech Mutli-Stream Input

**Figure 8:**
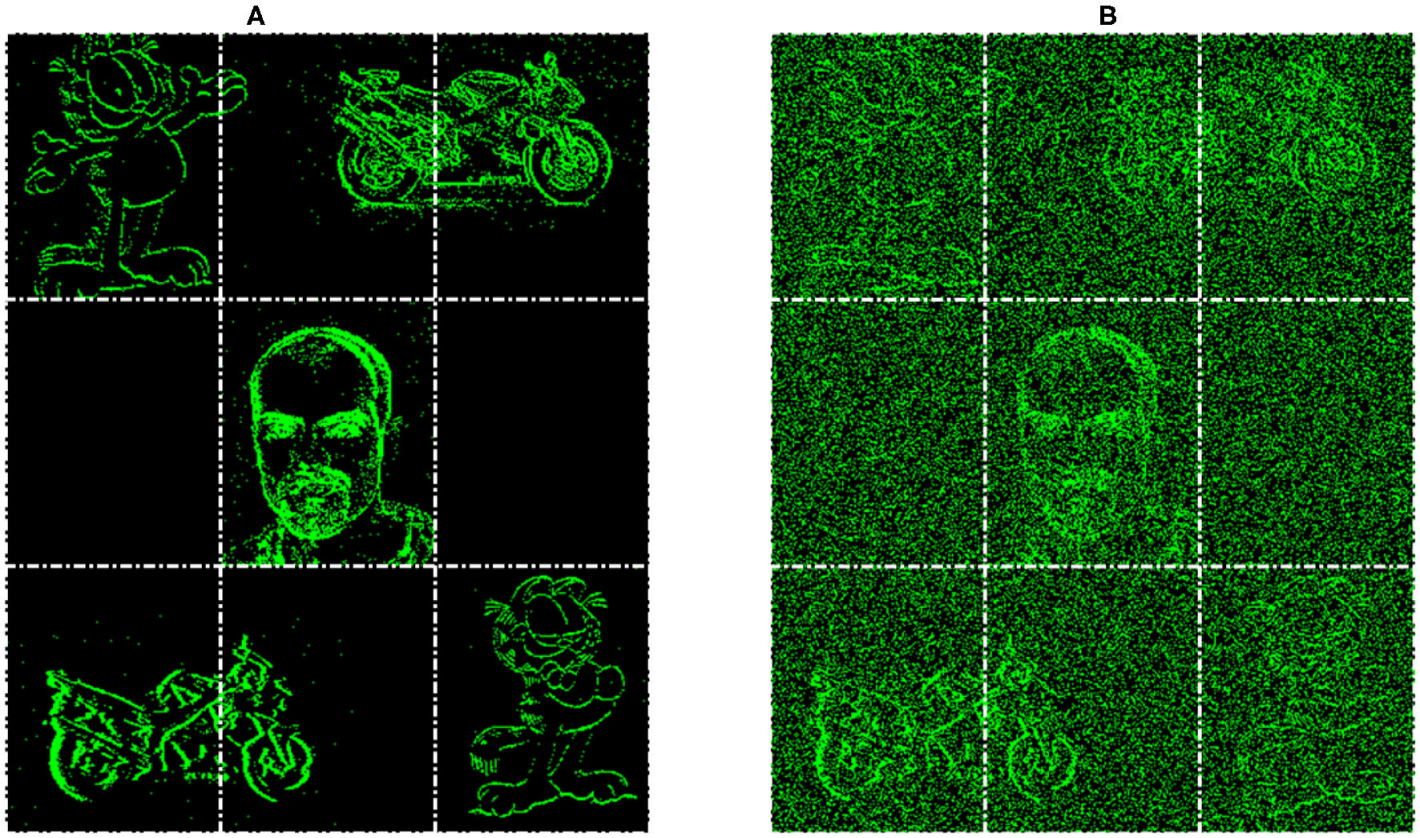
Example of input for the Multi-Stream Input without noise **(A)** and with noise **(B)**, both with extra gridlines indicating the 3 × 3 grid which determines the initial location of the inputs.

Building upon the results gathered from the successful process in section 4.1, this experiment looks at how the system would deal with multiple input streams. This allows the network to demonstrate the segmentation ability in the face of multiple distractors and spatio-temporal Gaussian noise with an average PSNR of 18 dB, an example of the input with and without noise is shown in Figures 8A,B, respectively.  Figure 8 also demonstrates the layout of the new input image, which is based on the Face class subset, but is three times the size to make a 3 × 3 grid where each corner and the center will host an input stream. Each stream is presented for 300 ms (dictated by the recording length in the dataset) then some of the locations are changed and the next stream is played. The input streams illustrated in Figures 8A,B, consist of one face and two motorbikes for the known classes and two Garfield streams for the unknown, with Figure 8B demonstrating the affect of noise on the input. This gives an opportunity to display the asynchronous layer-wise spike propagation once thresholds have been surpassed, while also offering an insight into how an SNN reduces computational throughout with this thresholding value.

**Figure 9:**
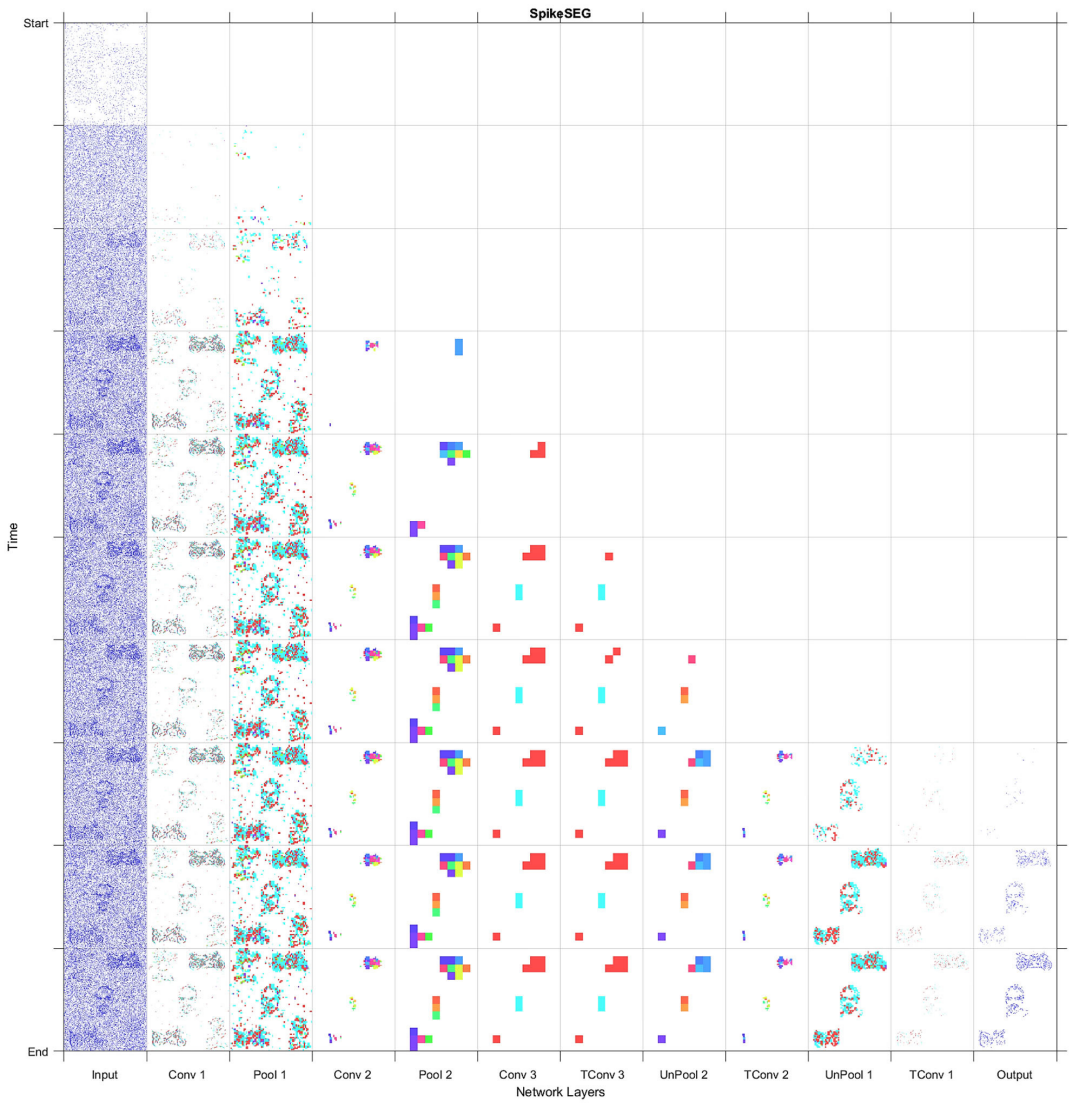
Full Layer-wise spiking activity for the system, showing the progression of spikes through the network encoding then decoding section into the segmentation output.

**Figure 10:**
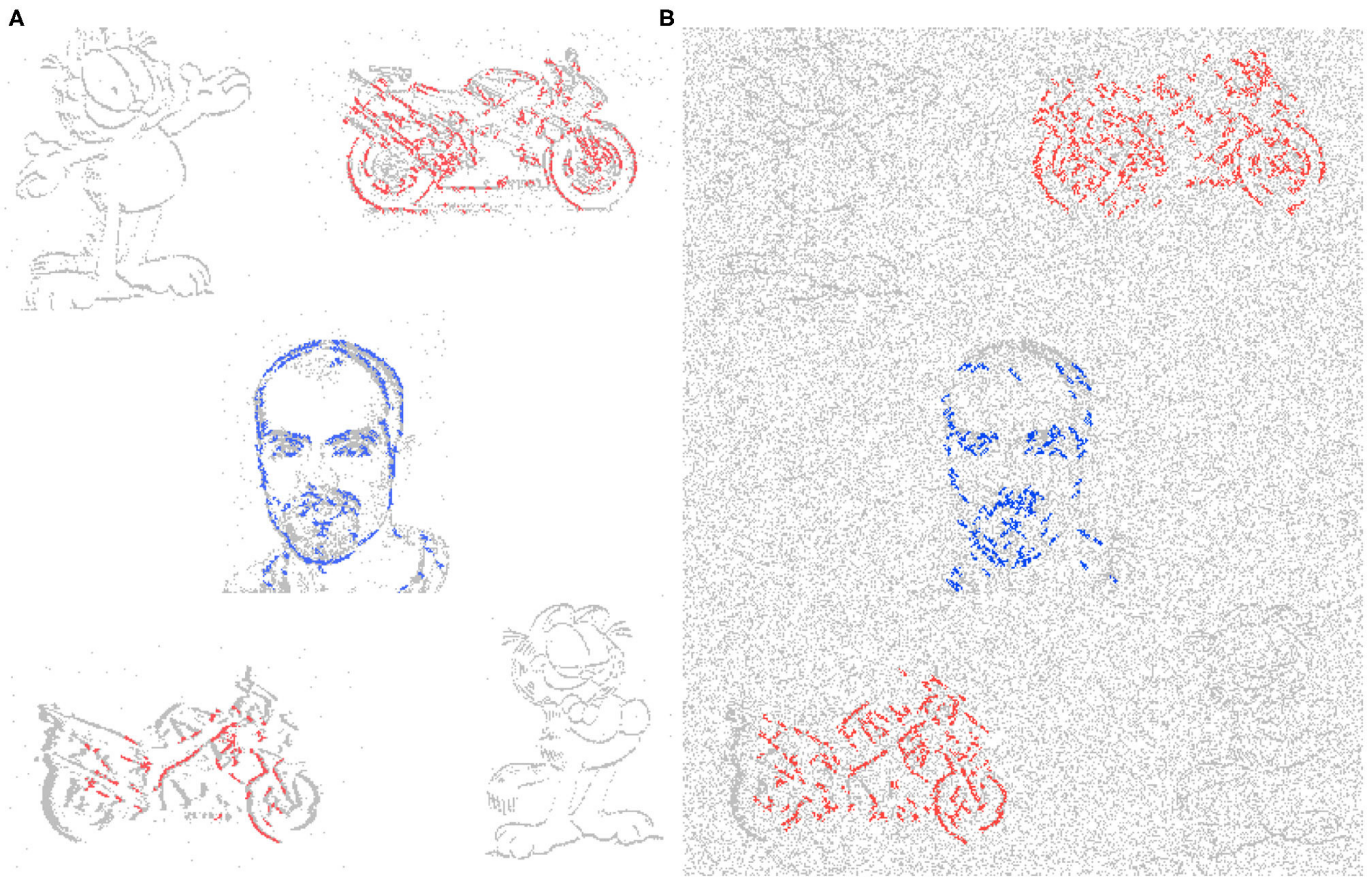
Segmentation overlays for the **(A)** Multi-Stream Input and **(B)** Multi-Stream Input with noise, including the classes Face, Motorbike, and Garfield from the N-CalTech dataset.

Figure 9 displays both this asynchronous throughput of activity and how the network reduces the numbers of computations, even when presented with noise and distractors, with the time axis showing an accumulation of spikes to ease with visibility. Figure 9 shows that by Conv 1 the added noise is mostly removed as it lacks any real structured shape, but the distractor, Garfield, remains and progresses onto Conv 2. During this layer though, due to its low saliency with any of the learned features for the classes of Face or Motorbike the distractor is removed from the processing pipeline. This leaves only the two known classes, which then progress onto the Conv3 layer, then through the decoder layers to the output where they are successful segmented. When testing the multi-stream input without any noise the accuracy and IoU value is identical to the single stream instance at 96.8 and 81%. Then with added noise this value sees a slight reduction to 95.1 and 79% for accuracy and IoU, these results are also shown in Table 1. The decreases being attributed to the noisy pixels directly contacting or occurring within the class boundary, as the network has no real way to discern this noise from actual data. This is clearly shown within the segmented output comparisons shown in Figure 10, where the noiseless output (A) and the noisy (B) show considerable difference in their respective segmentations with far more diagonal lines present in the noisy output (B) in comparison to (A). This outcome could have been predicted and will be highlighted in section 4.3 as the first layer of the network has a larger feature representation for the diagonal line when compared to the horizontal and vertical lines, with more pixels allocated to representing the diagonal lines rather than horizontal and vertical, due to the larger variety of edges this feature had to represent. Meaning relatively with the same threshold the diagonal feature is more likely to be activated than the horizontal and vertical.

**Figure 11:**
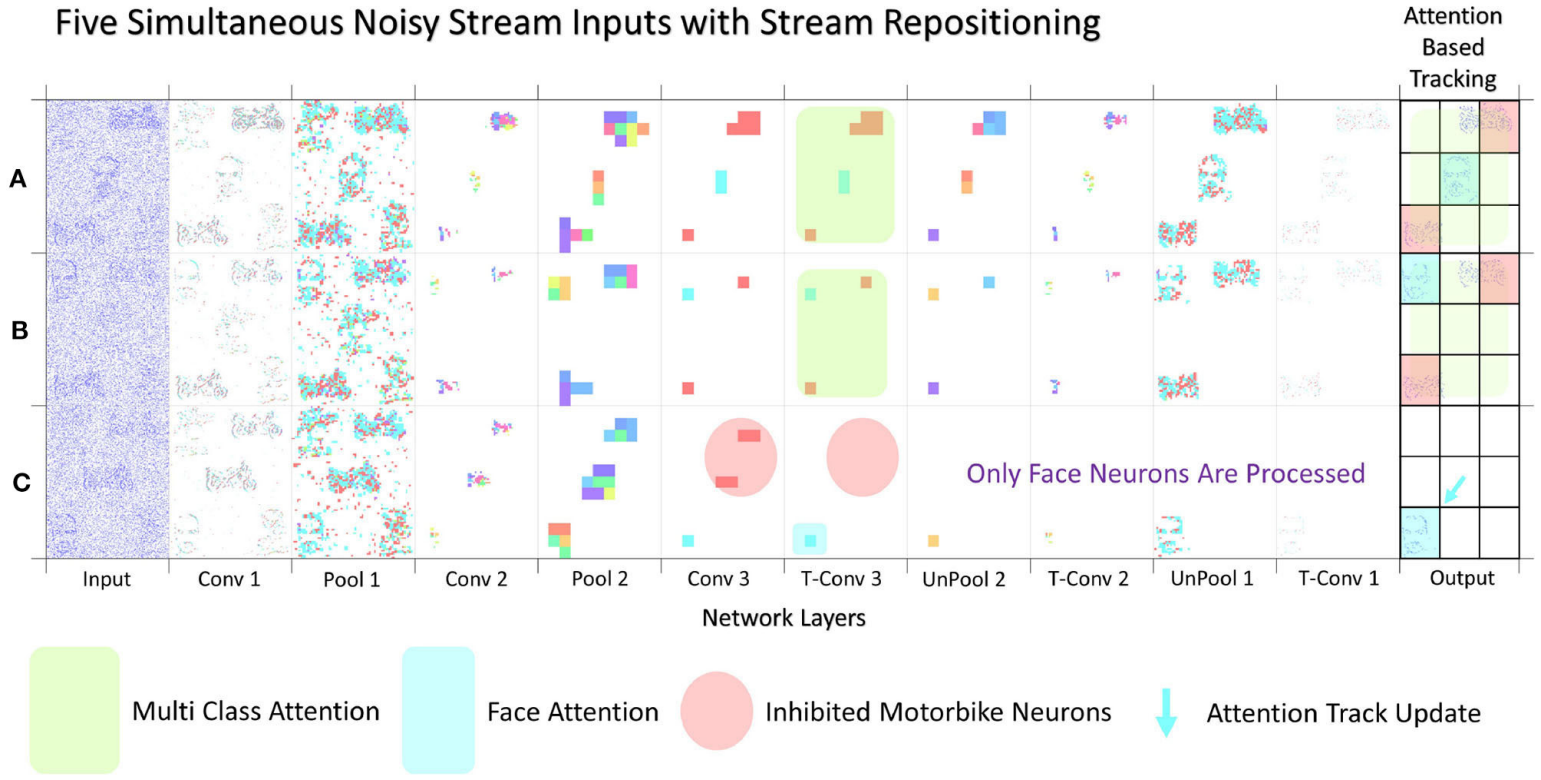
Image showing three separate multi input data streams. **(A,B)** both representing the full system layer-wise computations when no attention is selected, while **(C)** shows the layer-wise computation after the Face class has been selected as the attention of the network, thus enabling a simplification of the output and activating the action part of the system with a tracking controller update.

With the segmentation successfully output the spiking controller now has less spiking activity so should find it easier to be able to track a given class. The tracking starts once the user has made a selection of which class is to become the attention of the network. Experimentally this was tested by selecting the attention after two successful multi class segmentation examples where the stream inputs were repositioned. Figure 11 displays the outputs of the three inputs (A–C) with their subsequent paths to segmentation. Figure 11 shows that for inputs (A,B) the network is correctly segmenting the input and displaying an output with a highlighted segment displayed in the 3 × 3 grid. It is only in Figure 11C that the guided attention mechanism is triggered causing the inhibition of the other class in the propagation between layer Conv 3 and T-Conv 3. This feature is highlighted with the red circle showing which neurons are now no longer represented in the subsequent layer and thus no longer computed out to the segmentation, highlighting part of the efficiency in SNN. The last section of the diagram in Figure 11 highlights the attention of the network being drawn to the face located on the bottom left of the grid, which in the spiking controller would result in an output of left and down to ensure the face is located within the central region. The arrow within the Figure 11C also indicated the movement of the track update, which is based off the central region as within the previous two sequences the multiclass attention doesn't give a control output. This attention-based tracking update is delivered within 34 ms or 34 input steps, which with the 11 ms processing lag with each layer to propagate through the network results in a 31 ms delay within the 300 ms input stream. This may seem like a considerable amount of time, but as shown in both Figures 2, 9 due to the way the N-Caltech dataset was recorded, the first 30 ms of the recording contains very little information due to the lack of movement with the main concentration of spiking activity during the middle of each of the saccade movements. To test this the first 30 ms of events were removed from all the input streams which result in a reduction in track update to 15 ms and with the offset of 11 ms to progress through the network means a 4–5 ms latency to get from input to a control output if the processing could be done in real-time. However, even this latency is mainly from the initial delay in spiking activity within the network first layer, suggesting once running the latency would decrease. This would make it a highly competitive alternative or efficient middle ground between highly precise CNNs and low latency edge detection systems. Furthermore, the total number of average calculations represented by the images seen in Figure 11 is only ~9% of the total available calculations (equivalent CNN) due to the sparse nature of both the features and the SNN thresholding processing. Approximately 10% of capacity is used in the encoding process and ~5% in the decoding process, which is visualized in both Figures 9, 11.

#### 4.2.2. Tracking From Handheld NVS

**Figure 12:**
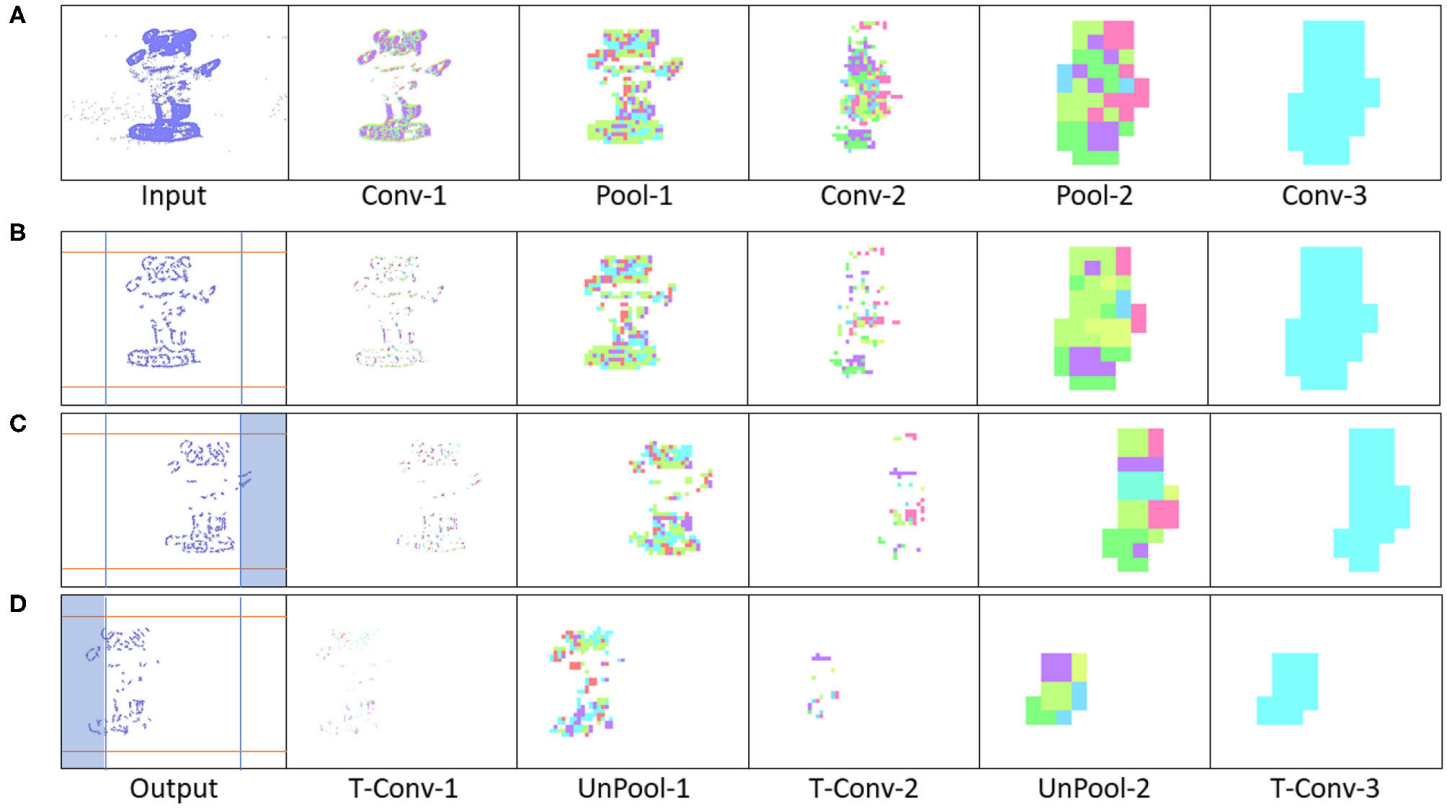
**(A)** Panda Input Image, **(B)** Panda being in the middle in the field of view, **(C)** Panda reaching rightmost boundary triggering a control action, **(D)** Panda reaching leftmost boundary triggering a control action.

For this section, the SpikeSEG network was retained to be able to identify a panda desk ornament and aims to better highlight the control and tracking aspects of the PUA system. The input stream recorded from DVS346 NVS has the panda start on the far left in the field of view then the camera pans to the left resulting in the panda being on the far right, with an example of the input images shown in Figure 12A. The results detail how well the segmentation would work within this example, with the extra complexity of 3D shapes and natural indoor lighting conditions. Overall the results of the 1 s test stream, show that only 60 ms (6%) of streaming footage failed to produce a segmentation output. This also occurs at the points where the least amount of movement of the camera happens, the turning points, subsequently producing fewer output spikes. Nevertheless, this results in no actual loss in tracking accuracy as the panda object stayed within the previous segmentations IoU bounding box. Furthermore, the IoU for this test stream was 75%, shown in Table 1, perhaps lower than expected given the high level of accuracy within the classification/segmentation process. This is illustrated in Figure 12A where the middle section of the panda is not well-resolved by the sensor, meaning on occasion the segmentation output was only of the top or bottom section. Figures 12B–D also show the full system process for the two different control outputs of moving left (D) and right (C), that is when the segmentation area enters the proximity of the spike counter at the edges of the output image. Within Figure 12D there is also an example of how the system overcame a background object that could have affected simpler approaches, as originally the input image had a background object on the right hand side of the image. Due to the feature extraction and segmentation, the background object was unable to influence the controller which without the Understanding-based segmentation would have had spiking activity in both left and right spike counters.

### 4.3. Robustness and Interpretability

This section highlights two key features of utilizing an SNN approach for this framework, the first is system robustness, especially that pertaining to Perception and Understanding (the sensor and processing) and how that affects the Actions of the system. The second feature is that of interpretability something that is not often not associated with CNN type approaches.

#### 4.3.1. Robustness

**Figure 13:**
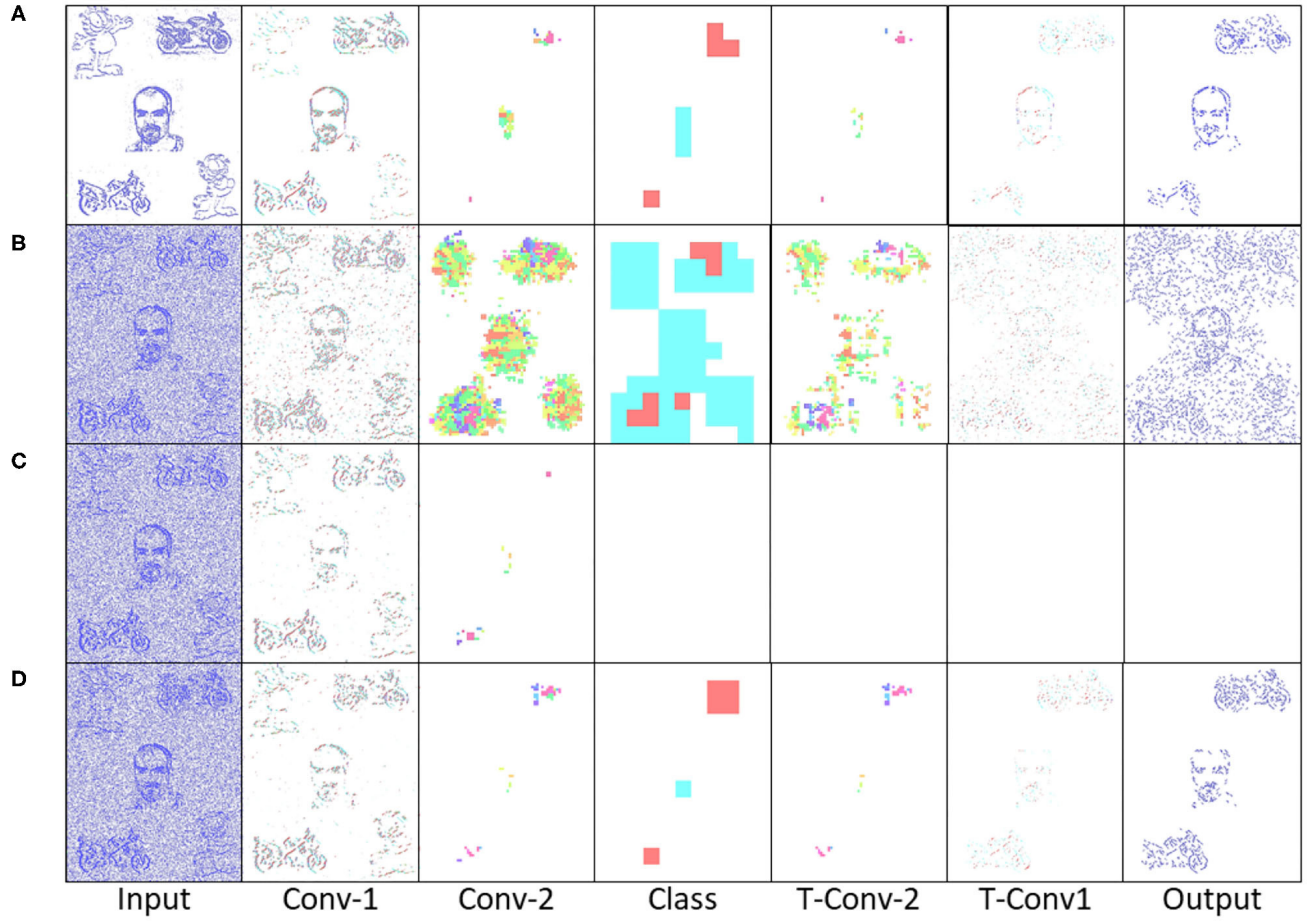
Highlighting the Robust noise suppression with the Pre-Empt then Adapt Thresholding mechanism. **(A)** Showing no noise input as reference, **(B)** Showing noisy input without PEAT active, resulting in noisey output, **(C)** Pre-Emptive Thresholding causing over suppression of neurons resulting in no output, **(D)** Shows the input to output with PEAT active, suppressing the noise but allowing the useful data to pass through.

The added robustness of the PUA approach comes from the Understanding section within the PEAT (Pre-Empt then Adapt Thresholding) mechanism. As mentioned in section 3.3.2, the buffering of input spikes allows a spike counter to be implemented, allowing a pre-emptive rather than reactive approach to the thresholding within the network. Permitting synaptic scaling homoeostasis to increase the threshold values on all layers, ensuring noisy or adversarial inputs are mitigated first. Subsequently, if the spike level persists the threshold levels using an intrinsic homoeostasis may be adapted. An example of this system at work is illustrated within Figure 13, with (A) showing a multi-stream input with no noise, then the input is corrupt with noise in (B–D) showing the resulting effects of the noise throughout the system with and without the PEAT mechanism active. The PUA framework implements the regime that no output is better than an incorrect output, especially when the input is degraded due to noise or adversarial sensor values. This robustness features is highlighted in the output of Figure 13B which is incorrect and if passed to the controller could cause an undesired response, meanwhile in Figure 13C the PEAT is seen to allow the network to threshold the noise level resulting in no segmentation output. Incidentally, Figure 13D could be the adaptive outcomes of both approaches (B,C), it is just intermediate control output suppression that adds an extra level of robustness to the system.

#### 4.3.2. Interpretability

**Figure 14:**
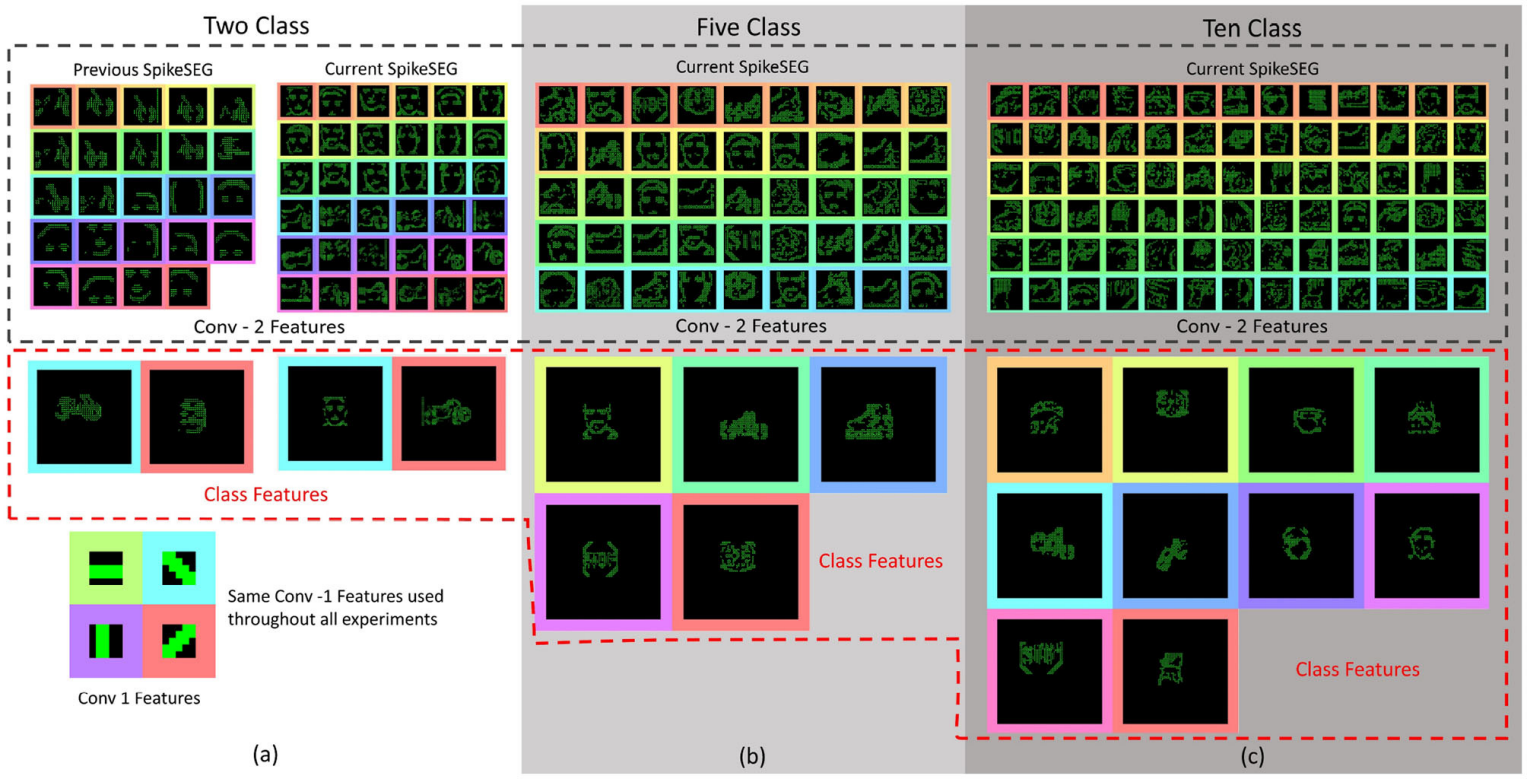
**(a)** Features map representations of the convolution layers, with coloring to match the latent space representation from the two class experiment, showing prior and current results of the Conv-2 features and Class features. The Figure also shows a selection of features from both the Five Class **(b)** and Ten Class **(c)** experiments. Top half showing the Conv-2 features and the bottom showing the Class Features. **(a)** Classes shown in Class Features are Motorbike-Face then Face-Motorbike for the previous and current results. **(b)** Classes shown in Class Features order are: Face, Motorbike, In-line Skate, Stop Sign, and Watch. **(c)** Classes shown in Class Features order are: Stegosaurus, Watch, Cup, In-line Skate, Motorbike, Revolver, Camera, Face, Stop Sign, and Windsor Chair.

The interpretability of a system is often overlooked when values of accuracy or precision appear to be high. But understanding or gaining some insight into how the system got to an answer could be a valuable advantage for SCNN compared to conventional CNNs. As SCNN trained using STDP happen to produce a sparse feature variation of typical CNN outputs, the SCNN results in features that are more akin of those from contour matching papers (Barranco et al., 2014) while CNNs typically take on features that resemble textures (Olah et al., 2017). These texture maps are often hard to interpret, although modern approaches have found ways to highlight the most salient parts of an input with reference to these texture maps. Nevertheless, it is still often difficult to predict how the system might react to an unknown input. The features that were learned for the testing of the N-Caltech dataset used within this work is shown in Figure 14. Figure 14A, illustrates the differences between the previous version of the model and the current implementation with PEAT and pruning improving the feature extraction, using the same Conv-1 features representing simple edge detection structure of horizontal, vertical and two diagonal lines. Figure 14A then shows the mapping those features onto the weights of the Conv-2 resulting in the features that resemble shapes and objects before the classification stage in Conv-3. It can be seen that half of the 36 features in Conv-2 relate to the Face class and the other half the Motorbike, with these features helping to build up the classification layers with two features either Face or Motorbike. This image helps to explain what the network has learned and how it appears to be looking for contour like shapes to help it distinguish between inputs. Along with this insight into how the network operates, it also allows the user to perhaps understand why it might not always give the correct answers. Similar to how if creating a system using hand-crafted contours features, you would understand the limitation this allows a similar understanding to be had. This could allow manual manipulation of features or manual pruning throughout the training if the user happens to have expert knowledge of the task, bringing neural networks closer to known computer vision-based techniques, which could provide an interesting overlap, especially in the robotics domain.

In order to perceive how the additional classes affects the interpretability of the system Figures 14B,C highlights a sub-selection of the features within the 5 and 10 class models. This highlights how the interpretability is still there for some of the features while others have become more difficult to understand, perhaps due to overlapping features from two classes. Overall, Figures 14B,C highlight how reviewing of the features within a Spiking Neural Network can help to gain understanding about parts of the network, with the classification layers features representing each of the 5 and 10 classes. The visualizations help to explain why certain classes might struggle vs. others due to similar sub classification features.

## 5. Discussion

The understanding method shown in this work details an unsupervised STDP approach. To fully utilize the spiking nature of the processing it is paired with the perception method of spiking input sensor. Together this perception understanding pair can successfully semantically segment up to 10 classes of the N-Caltech dataset. The output of this process is a spiking grid indicating the location of the class within the scene, which can be interpreted by the action system to allow the objects to be followed if attempting to leave the field of view.

The full PUA process is completed in a spiking and fully convolutional manner. This ensures all calculations are either spiking or spike counting. Allowing the network to maintain the temporal and processing advantages, along with the asynchronicity associated with neuromorphic vision sensors, from input to output. However, this method of processing is not without its drawbacks, as there is an overall decrease in accuracy associated with this adding of extra classes. That perhaps indicates the limitation with this unsupervised method in terms of problem scaling. For instances with the 100 classes available within the N-Caltech dataset, the system would only be able to learn the most common features that occur within each class, but only if they present a large enough variance. That is it will only learn common class features as long as they look different enough from the other classes. Which is essentially what can be seen happening with the 5 and 10 class experiments visualized in Figures 14, 7C. Figure 7C highlights that even with a high inter class variance the kernels sometimes learn differentiating features from all other classes, while other times learns features that are an amalgamation of two or more classes. The 5 class experiment displays this most prominently with the Bike and In-Line Skate classes, as there are similarities between the outline shape of both objects.

Nevertheless, this ability to find most common features that express the highest variance from others, is both the limitation and strength of this STDP approach. Limiting in that this approach might not scale to larger datasets, but a strength in that it made the network asynchronous, adaptable, computational sparse and visually interpretable. This highlights that the STDP method used might not be suitable for all problems, but serves as a indication of the benefits if the problem is appropriate. This work demonstrates that STDP alone can be used to find the most common features of a dataset. Which in turn, can be used to successfully perform image classification and semantic segmentation. However, a further learning rule to help focus on more discriminative features, such as R-STDP (Izhikevich, 2007; Legenstein et al., 2008; Mozafari et al., 2018) would be a useful extension. This could help in tackling the main issue of inter to intra class variance differentiation. This could allow not only the most common feature to be discovered, but the most common discriminative feature.

## 6. Conclusion

We proposed a new spiking-based system, the Perception Understanding Action Framework, which aims to exploit the low latency and sparse characteristic of the NVS in a fully neuromorphic asynchronous event driven pipeline. Using the understanding gained through the SpikeSEG segmentation, the network is able to detect, classify and segment classes with high accuracy and precision. Then from this understanding, the system makes a more informed decision about what action is to be taken. In this context, the framework was able to show a semantic class tracking ability that combines feature extraction capability of CNNs and low latency and computation throughput of line and corner detection methods. The framework also explores the unique benefits that can be gained through utilizing SNNs with interpretability and robustness, with the use of thresholding algorithms and sparse feature extractions. The PUA framework also shows off the unique attention mechanism, emphasizing how simple local inhibition rules when combined with an encoder decoder structure; this can help reduce the computation overhead of the semantic segmentation process. This research highlighted the series of benefits when utilizing a fully neuromorphic approach with a pragmatic engineering and robotics outlook, looking at the biologically inspired mechanisms, features and benefits, then combining them with modern deep learning-based structures.

## Data Availability Statement

The raw data supporting the conclusions of this article will be made available by the authors, without undue reservation.

## Author Contributions

PK contributed to the framework and experimental work. PK, GD, and JS contributed to the paper writing. All authors contributed to the article and approved the submitted version.

## Conflict of Interest

GM was employed by the company Leonardo MW Ltd. The remaining authors declare that the research was conducted in the absence of any commercial or financial relationships that could be construed as a potential conflict of interest.
